# High-Density SNP-Based Association Mapping of Seed Traits in Fenugreek Reveals Homology with Clover

**DOI:** 10.3390/genes11080893

**Published:** 2020-08-05

**Authors:** Mustafa M. H. Abd El-Wahab, Maha Aljabri, Mohamed S. Sarhan, Gamal Osman, Shichen Wang, Mahmoud Mabrouk, Hattem M. El-Shabrawi, Ahmed M. M. Gabr, Ahmed M. Abd El-Haliem, Donal M. O’Sullivan, Mohamed El-Soda

**Affiliations:** 1 Department of Agronomy, Faculty of Agriculture, Cairo University, Giza 12613, Egypt; mustafamh2003@yahoo.co.uk (M.M.H.A.E.-W.); mahmoudm.mabrouk92@gmail.com (M.M.); 2 Department of Biology, Faculty of Applied Sciences, Umm Al-Qura University, Makkah 21955, Saudi Arabia; Myjabri@uqu.edu.sa (M.A.); geosman@uqu.edu.sa (G.O.); 3 Research Laboratories Centre, Faculty of Applied Science, Umm Al-Qura University, Makkah 21955, Saudi Arabia; 4 Environmental Studies and Research Unit, Cairo University, Giza 12613, Egypt; m.sabrysarhan@gmail.com; 5 Agricultural Genetic Engineering Research Institute (AGERI), ARC, Giza 12915, Egypt; 6 Genomics and Bioinformatics Service Texas A&M AgriLife Research, Amarillo College Station, Amarillo, TX 77845, USA; Shichen.Wang@ag.tamu.edu; 7 Plant Biotechnology Department, National Research Center, Giza 12622, Egypt; helshabrawi73@yahoo.com (H.M.E.-S.); a_m_gabr2@yahoo.com (A.M.M.G.); 8 Plant Physiology, University of Amsterdam, Swammerdam Institute for Life Sciences Amsterdam, 1098 XH Amsterdam, The Netherlands; amabdelhaliem@gmail.com; 9 School of Agriculture, Policy and Development, University of Reading, Whiteknights, Reading RG6 6AR, UK; d.m.osullivan@reading.ac.uk; 10 Department of Genetics, Faculty of Agriculture, Cairo University, Giza 12613, Egypt

**Keywords:** fenugreek, population structure, ddRAD-sequencing, SNP markers, association mapping, homology

## Abstract

Fenugreek as a self-pollinated plant is ideal for genome-wide association mapping where traits can be marked by their association with natural mutations. However, fenugreek is poorly investigated at the genomic level due to the lack of information regarding its genome. To fill this gap, we genotyped a collection of 112 genotypes with 153,881 SNPs using double digest restriction site-associated DNA sequencing. We used 38,142 polymorphic SNPs to prove the suitability of the population for association mapping. One significant SNP was associated with both seed length and seed width, and another SNP was associated with seed color. Due to the lack of a comprehensive genetic map, it is neither possible to align the newly developed markers to chromosomes nor to predict the underlying genes. Therefore, systematic targeting of those markers to homologous genomes of other legumes can overcome those problems. A BLAST search using the genomic fenugreek sequence flanking the identified SNPs showed high homology with several members of the Trifolieae tribe indicating the potential of translational approaches to improving our understanding of the fenugreek genome. Using such a comprehensively-genotyped fenugreek population is the first step towards identifying genes underlying complex traits and to underpin fenugreek marker-assisted breeding programs.

## 1. Introduction

Fenugreek (*Trigonella foenum-graecum* L.) is a small-seeded annual dicotyledonous legume that belongs to the family Leguminosae (Fabaceae). The genus *Trigonella* belongs to the Trifolieae tribe and the Trigonellinae subtribe that includes other several genera such as *Trifolium*, ***Melilotus***, and *Medicago* [1,2]. Fenugreek is a famous aromatic spice derived from the dry seeds and green leaves and has been used since ancient times in Roman, Chinese, Indian, and Egyptian history as a human food and herbal medicine. The ancient Egyptian medical papyrus of herbal knowledge dating to circa 1500 BC, known as the Ebers Papyrus, described its medical use and benefits. In the modern food industry, it can be used as a supplement for wheat and maize flour in bread making as a source of flavor, color, and to modify the texture of food materials [3,4].

Fenugreek (*Trigonella foenum-graecum* L.) is a small-seeded annual dicotyledonous legume that belongs to the family Leguminosae (Fabaceae). The genus *Trigonella* belongs to the Trifolieae tribe and the Trigonellinae subtribe that includes other several genera such as Trifolium, Melilotus, and Medicago [1,2]. Fenugreek is a famous aromatic spice derived from the dry seeds and green leaves and has been used since ancient times in Roman, Chinese, Indian, and Egyptian history as a human food and herbal medicine. The ancient Egyptian medical papyrus of herbal knowledge dating to circa 1500 BC, known as the Ebers Papyrus, described its medical use and benefits. In the modern food industry, it can be used as a supplement for wheat and maize flour in bread making as a source of flavor, color, and to modify the texture of food materials [3,4].

As a self-pollinated plant, selecting single new lines has been proven as a powerful breeding approach for selecting highly heritable quantitative traits such as seed size, and seed color [5,6,7,8]. Fenugreek infloresences can produce 2–8 pods, each containing 10–20 small and hard seeds. Seed size ranges from 4.01 to 4.19 mm (length), and 2.35 to 2.61 mm (width), and in seed colors ranging from dull yellow, brownish yellow, olive green, brown, cinnamon, and lighter green [9,10,11]. Very little effort has been made to estimate the genetic variability among fenugreek genotypes in recent years in spite of the advancement in the sequencing technologies. However, several studies have investigated fenugreek genetic diversity using various traditional genetic markers [10]. For example, 17 accessions were evaluated using 14 ISSR and 22 RAPD markers [12]. Another study investigated 90 genotypes using 13 SSR and 49 RAPD markers [13]. Recently eight landraces were examined using six SRAP primers combination [14]. However, these studies lacked statistical power (due to the low number of genotypes and low marker density) to lay the foundations for mapping complex quantitative traits. 

The traditional approach to genetically dissect such complex traits and to identify the underlying quantitative trait loci (QTL) or genes is by using the progeny of selected crosses such as recombinant inbred lines, backcrosses, or double haploid populations. However, this approach suffers from the limited variation existing in the parents and the time required to create such populations. Therefore, the use of the alternative approach known as association mapping (AM), relying on linkage disequilibrium (LD) between polymorphic molecular markers and the causal variants in a large number of individuals, has become commonplace. The advantage of AM over traditional QTL mapping is the use of already-existing naturally evolved and adapted genotypes with wider genetic variation, eliminating the need to generate new mapping populations. This criterion makes neat use of the historical recombination accumulated over hundreds or thousands of generations in a large number of diverse genotypes. To detect such crossing-overs, those genotypes need to be densely genotyped to obtain high statistical mapping resolution and to identify single nucleotide polymorphisms (SNP) associated with the examined trait [15,16]. To achieve this goal, several sequencing technologies such as restriction site-associated DNA sequencing (RAD-seq) [17], which uses one restriction enzyme to randomly generate genomic DNA fragments, have facilitated genotyping many more SNP markers than was previously feasible. RAD-seq can be used to study plants without reference genomes. However, it reveals a significant loss of the data due to sequence read errors [18]. To address these shortcomings, the double digest RAD-seq (ddRAD-seq) [18] technique, which uses two restriction enzymes to digest the genomic DNA, was developed. The resulting fragments undergo adaptor ligations, precise size selection, and a very small fraction of the fragments are sequenced [18]. 

Very little information is available about the fenugreek genome. Most available studies focused on identifying genes involved in the biosynthesis of diosgenin using de novo transcriptome sequencing [19] and next-generation sequencing (NGS) of representational difference analysis (RDA-NGS) [20]. Two very recent studies used comparative transcriptome analysis [21], and qRT-PCR [22]. Some gaps in our knowledge of fenugreek genome structure and function could potentially be filled by leveraging homology comparisons with known genomes, similar to the recent studies in the Trifolieae tribe of the Fabaceae family between *Medicago truncatula* (barrel clover) and *Trifolium repens* L. (white clover) [23], and between *Trifolium pretense* and *Trifolium medium* (zigzag clover) [24]. 

To our knowledge, no previous study has examined the population structure and association mapping or homology analysis using fenugreek germplasm genotyped with a large number of SNPs. Therefore, we used a local collection of 112 genotypes collected from different locations in Egypt and genotyped at high density using the ddRAD-seq technique. 

## 2. Materials and Methods

### 2.1. Fenugreek Genotypes Collection

**Figure 1:**
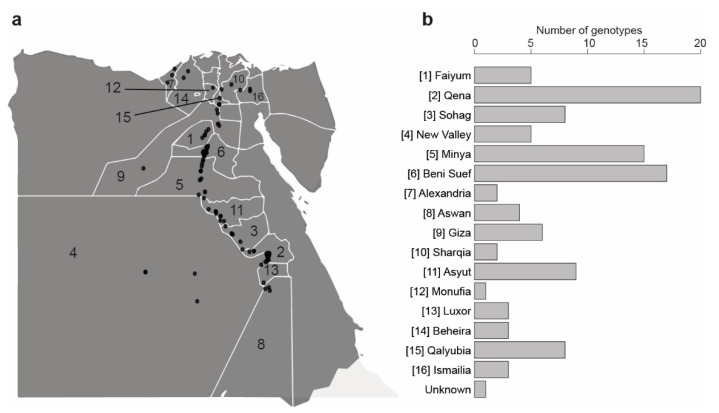
GPS coordinates of the locations where the fenugreek genotypes were collected; please refer to Table S1 for further details; (**a**), GPS locations plotting (numbers indicated in the map represent the Egyptian governorates from which genotypes were collected and correspond to those in the panel (**a**)); (**a**), horizontal bar-plot showing the number of genotypes of each Egyptian governorate.

To our knowledge, there are no certified accessions collected in the Egyptian gene bank. Therefore, we have collected seeds of 112 fenugreek genotypes directly from local farmers in all governorates that produce fenugreek (Figure 1 and Table S1). The largest numbers of genotypes were collected from the main producing governorates, i.e., Qena (20 genotypes), Beni-Suef (17 genotypes), Minya (15 genotypes), Asyut, (nine genotypes) and Sohag (eight genotypes). Each genotype was collected from a different farmer, its seeds are homogenous and show different phenotypes from other genotypes. These new collections of farmer-maintained genotypes were supplemented with seven ex situ conserved genotypes obtained from the Genetics Resource Center (GRC), Qalyubia, Egypt. The GRC is a small and local initiative by the Egyptian researchers to collect different plant genotypes.

### 2.2. DNA Extraction and Library Preparation for Sequencing

Genomic DNA was extracted from the seeds of 112 Fenugreek genotypes using Quick-DNA plant/seed Miniprep kit (www.zymoresearch.com). DNA concentration was measured using NanoDrop (One/OneC, Model: ND-ONE-W, NanoDrop, Thermo Scientific, Waltham, MA, USA). One hundred micrograms of DNA per sample in 96 well plates were digested in 1× NEB Cut Smart Buffer with *Eco*RI and *Mbo*I (NEB) at 37 °C for 4 h. Following a 20 min 80 °C enzyme inactivation, samples were held at 12 °C until ligation with T4 DNA Ligase (NEB) and adapters containing 1 of 48 unique barcodes and Illumina-compatible P5 sequences coupled to an *Eco*RI overhang and Illumina-compatible P7 sequences coupled to the *Mbo*I overhang. Plates were incubated 8 h at 16 °C and heat inactivated at 80 °C for 20 min. Samples were then pooled in three pools of 40, 38 and 34 samples respectively and mixed with EDTA and ethanol precipitated. Pellets were re-suspended in EB, purified with PCR Purification columns (Qiagen, Germantown, MD, USA) and further cleaned up with one volume of AMPure XP beads. One to three µg DNA was subjected to Pippin Prep size selection on a 2% dye-free agarose gel with internal size markers aiming for 350–500 bp inserts. Recovered samples were cleaned with AMPure XP beads and subjected to a pre-selection PCR (PreCR) in which a biotinylated forward primer and unique indexed reverse primers were used to amplify and tag desired DNA fragments. PCR products were cleaned up with Qiagen PCR purification columns and 1X AMPure XP beads as before. DNA fragments, with biotin at the 5′ ends, only were selected using Dynabeads M-270 Streptavidin coupled magnetic beads (ThermoFisher). Briefly, 50 µL of beads were mixed with up to 2000 ng of each pool and incubated for 20 min at RT. Bead/DNA complexes were captured and washed several times, then resuspended in 50 µL 1× SSC and heated at 98 °C for 5 min then placed on a magnet and supernatant removed as soon as possible. This elution was repeated, and the final supernatants were cleaned up with Qiagen PCR columns. The eluted ssDNA was quantified and diluted to 1 ng µL^−1^ with EB. A final PCR was performed on 10 ng of input DNA using P5 and P7 primers with only 8 cycles. Final PCR products were purified with 1× AMPure XP beads, quantified and assessed for quality on a Fragment Analyzer System (Agilent Technologies, Santa Clara, CA, USA). The samples were sequenced at the Texas A&M AgriLife Genomics and Bioinformatics Services on one lane of Illumina NovaSeq 6000 using a S4 XP sequencing kit. The raw sequencing reads are available through the NCBI BioProject number PRJNA648770, and the NCBI BioSample SAMN15647967. 

### 2.3. Bioinformatics and Statistical Analyses

We checked the raw reads for quality using FastQC [25,26]. Raw sequencing data were then processed using the dDocent pipeline v2.2.6 [27]. Briefly, the raw sequencing data were first processed with quality filter using the tool TrimGalore [28], which removes Illumina sequencing adapters, trimmed low-quality bases (Phred score < 20) on the end of reads and used an additional 5 bp sliding window to trim bases with average quality score below 10; then the quality filtered reads were mapped to the de novo assembly reference constructed with rainbow [29], using the BWA MEM algorithm with default parameters. Only reads with coverage depth above 3× and presented in more than 10% of the total samples were selected for de novo assembly. CD-HIT was used to cluster reference sequences by similarity of 86% [30]. Alignment files generated for each sample were then processed by the program FreeBayes [31], with parameters set as “–E 3 -q 10 -m 10”, to detect single nucleotide polymorphisms (SNPs) from the aligned reads.

### 2.4. Population Structure Estimation

Population structure was analyzed using the standard pipeline implemented in fastSTRUCTURE [32] and ΔK range values of 2–13. We further used the “*chooseK.py*” script to estimate the optimal number of components that explain the population structure, maximizing the marginal likelihood. Then, the admixture proportions of the individuals were visualized using the “*distruct.py*” [32]. To further validate the fastSTRUCTURE outputs, we constructed a phylogenetic tree using the matrix of 38,142 SNPs × 112 individuals.

### 2.5. Phenotyping and Association Mapping

Seeds were cleaned and placed on a white opaque sheet with a rigid sparse to guarantee to have single separated seeds. For each genotype, 15 homogeneous, in shape and color, and healthy seeds were selected and separated then represented in one picture with a resolution of 4632*2608 (width*height). A digital camera was fixed at 25 cm height and JPEG images with a resolution of 96 dpi were taken and analyzed using ImageJ software (National Institutes of Health, USA, https://imagej.nih.gov/ij/). Seed length and width were measured for every single seed and the mean value of the 15 seeds was used for association mapping. ImageJ generated three values of red (R), green (G), and blue (B) colors for each genotype that were used in the following equation [33] to calculate the final RGB color:

(1) RGB=(R×65536)+(G×256)+B

Broad-sense heritability was estimated for the raw data as the ratio between the genetic variance Vg, and the total phenotypic variance Vt, with Vt = Vg + Ve, where Ve is the environmental variation, i.e., the variance between replications of each genotype. For association mapping, a qualitative form of the three traits were used as shown in Table S2. Considering the data range, seed length was split into 2 groups and seed width and seed color were split into 4 groups. Association mapping was performed by the Genomic Association and Prediction Integrated Tool (GAPIT) package in R software [34] using mixed linear model (MLM) approach [35], Kinship matrix and principal components [34,36]. To correct for multiple testing, we used Bonferroni correction [37] and false discover rate (FDR) of α = 0.05 [38,39,40].

### 2.6. Homology Analysis to Predict Candidate Genes

To map possible candidate genes associated with seed length, width, and color, we used the online NCBI BLASTn tool and the non-redundant (NR) database (https://blast.ncbi.nlm.nih.gov/Blast.cgi) to search for sequences showing high homology to the contig sequences on which the significant SNPs associated with these traits were located. The identified top hit sequences were subsequently subjected to six-frame translation followed by a search for existing, conceptual, Open Reading Frames (ORFs). To predict the putative function of the genes represented by these ORFs, we scanned the corresponding protein sequences for conserved domains using the Simple Modular Architecture Research Tool (SMART) (http://smart.embl-heidelberg.de/). To investigate whether the identified genes, in which the SNPs are located, are transcribed, we collected public, raw, fenugreek RNAseq data from the Sequence Read Archive (SRA) at NCBI (https://www.ncbi.nlm.nih.gov/sra) and used it to construct either a genotype-specific (from one SRR file representing a single fenugreek accession) or a consensus (from multiple SRR files representing multiple fenugreek accessions) de novo transcriptome assembly. For that, we made use of the software package Trinity in combination with Trimmomatic for filtering and trimming the reads and then used local BLAST (BLAST+ 2.10.0, NCBI) to search for mRNA transcripts matching our contigs.

## 3. Results 

### 3.1. Sequencing Quality 

Overall, we obtained 425,741,340 pair-end 125 bp reads, with average of 3.6 million reads per sample. After quality trimming and filtering using TrimGalore, ~2.6% of the reads were removed prior to de novo assembly using the dDocent assembler (ddocent.com). A total of 83,532 unique contigs were assembled and further used as reference for mapping the reads. Among these contigs, FreeBayes reported >2M raw variations that were further filtered to minimize the calling of false SNPs due to sequencing error, paralogs, or artifacts from library preparation. The raw variant call file (VCF) was filtered using vcftools v0.1.15 [41] with a minimum quality score of 30, minimum genotype depth set to 3 reads, no more than 0.2% missing data per SNP (except for genotypes G46, G89, G68, G19, G67, G64, G44, G20, G31, G126, G48, G57, and G45), and minimum mean depth of coverage (DP) of 20. Only bi-allelic SNPs with a minimum minor allele frequency (MAF) of 0.05 were retained for downstream analysis. Finally, after the previous filters had been applied, 38,142 SNPs remained in the final dataset.

### 3.2. Population Structure and Genetic Diversity

**Figure 2:**
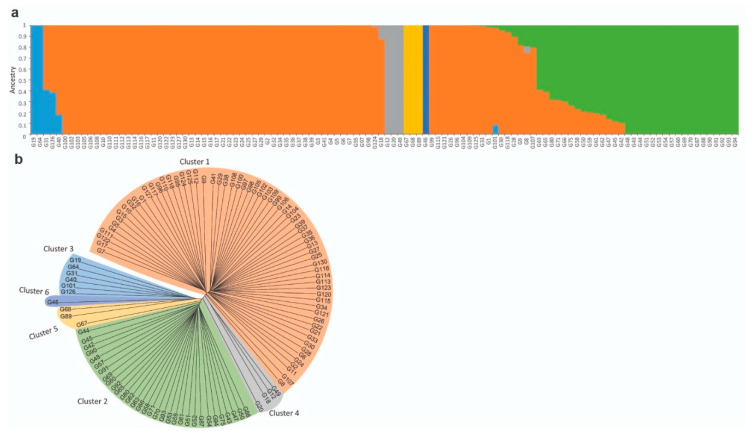
Population structure analysis showing the admixture proportions of the 112 fenugreek genotypes (**a**), and the SNP-based phylogenetic analysis (**b**). In both panels, genotype IDs (in the format G+number) match those presented in Table S1.

The 38,142 generated SNPs of the 112 tested fenugreek genotypes were used as an input for the fastSTRUCTURE software [32], to build an admixture model and to reveal the population structure. Testing the number of genetic clusters (K) within a range of 2–13, at Hardy–Weinberg (HW) equilibrium, the model complexity that maximizes the marginal likelihood was suggested as K = 6, while the model components that best explain structure in the data were suggested as K = 2. Additionally, the analysis revealed strong genetic structure in most of the genotypes and a moderate degree of admixture within other genotypes which appeared to be independent of their geographic origin (Figure 2a). Results obtained from the SNP-based phylogenetic analysis were consistent with the population structure, displaying two main clusters and six sub-clusters (Figure 2b). The first main cluster consisted of 66 genotypes showing strong genetic structure. The second main cluster included the other 46 admixed genotypes that were further divided into five different sub-clusters. 

### 3.3. Association Mapping

**Figure 3:**
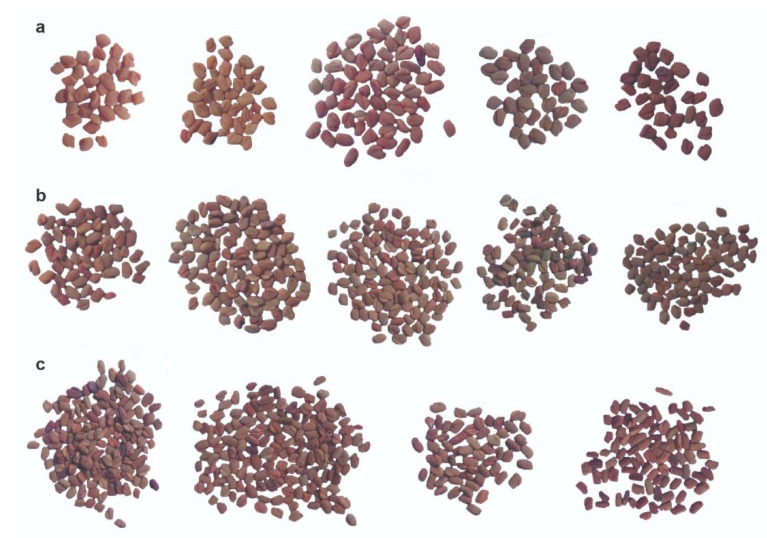
Images of the observed variation in seed length, width and color. Raw (**a**) include large seed size, raw (**b**) include medium seed size, and raw (**c**) include small seed size, all ascending from left to right based on color darkness.

A high level of variation was observed among genotypes for seed length, with a heritability of 0.53, and seed width, with a heritability of 0.51 (Figure 3 and Table S2). Minimum values of 1.80 and 1.03 mm and maximum values of 3.18 and 2.31 mm were recorded for seed length and width with mean values of 2.31 and 1.65 mm, respectively. For seed color, three main categories were observed, i.e., yellow, light-brown and dark-brown (Figure 3).

**Figure 4:**
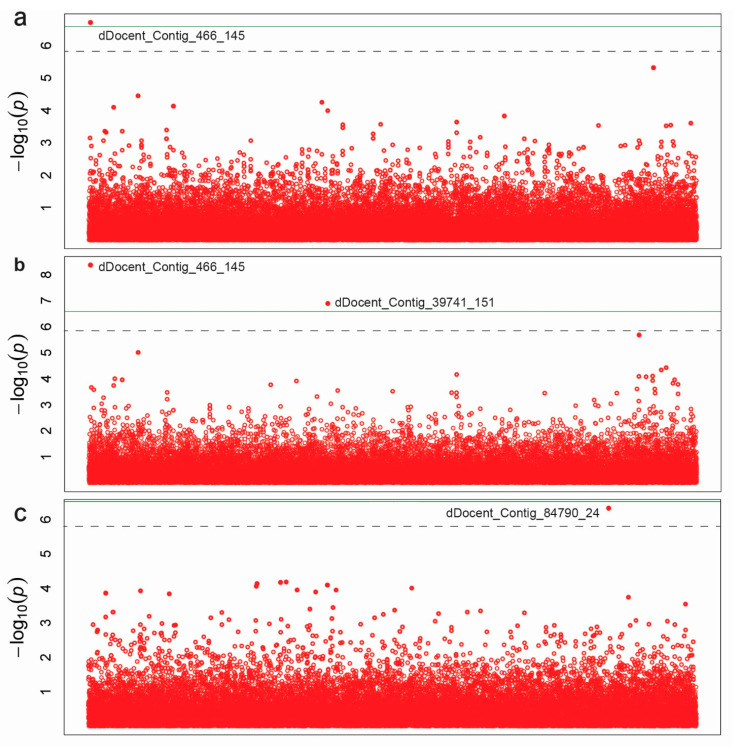
Manhattan plots representing association mapping for the 112 fenugreek genotypes using 38,142 SNPs of seed length (**a**), width (**b**), and color (**c**) arranged in random order (x-axis). The green horizontal solid line represents Bonferroni correction threshold at 6.5. The dashed gray line represents false discovery rate at 5.9.

SNP-based association mapping was performed using a mixed linear model (MLM) excluding rare alleles with minor allele frequency (MAF) < 5%. The MLM included population structure (Q), and kinship matrix (K) to avoid spurious associations. The −log 10(*P*) association detection threshold was set to 6.5 and 5.9 based on Bonferroni correction, and FDR of α = 0.05, respectively. As shown by the Manhattan plots (Figure 4), the SNP dDocent_Contig_466_145 was significantly associated with seed length and width at −log10(*P*) values of 6.71 and 8.36, respectively (Table 1 and Figure 4), while the FDR for this SNP was α = 0.007 and 0.000. 

This SNP explained 39 and 46% of the seed length and width variation, respectively. The second significant SNP, dDocent_Contig_39741_151, was associated only with seed width with −log10(*P*) = 6.88, FDR of α = 0.002, and explained 38% of the seed width variation. In total, the two SNPs explained 84% of the seed width variation. For seed color, the SNP dDocent_Contig_84790_24 was associated at −log10(*P*) = 6.32, FDR of α = 0.016, and explained 28% of the seed color. The sequences of these three ddRAD markers are provided in Table 1. 

### 3.4. Homology Analysis to Predict Candidate Genes

BLASTn searches against the NR database at NCBI using the sequences of the three contigs (Table S3) indicated significant homology between the contig dDocent_Contig_466, on which the SNP dDocent_Contig_466_145 was mapped, and genomic sequences from *Medicago truncatula* and *Trifollium* spp. Similarly, the contig dDocent_Contig_84790, which contains the SNP dDocent_Contig_84790_24, was found to have homologous sequences in the same two species. In contrast, no BLAST hits were identified for the second contig, dDocent_Contig_39741, containing the SNP dDocent_Contig_39741_151. The identified top hit sequences from *M. truncatula* and *Trifolium* were subsequently subjected to six-frame translation followed by a search for existing, conceptual, ORFs. Surprisingly, both fenugreek SNPs, dDocent_Contig_466_145 and dDocent_Contig_84790_24, were aligned to open reading frames in *M. truncatula* and *Trifollium*. Domain search using the SMART tool identified a retrotransposon GAG domain, where the SNP dDocent_Contig_466_145 was aligned at the C-terminal end of this domain in both top sequences hits from *M. truncatula* and *Trifolium* spp. (Table S3). The GAG domain (Pfam: PF03732) is a relatively conserved domain found in several terminal repeat retrotransposons known as TR-GAGs [42]. Similarly, the SNP dDocent_Contig_84790_24 was aligned directly downstream of a retroviral integrase domain (Pfam: PF00665), which is a common catalytic domain in the “gypsy” type of retrotransposons. As TR-GAGs transposable elements (TEs) are known to be actively transcribed [42], we investigated whether the identified TE genes, in which the SNPs are located, are also transcribed. Using the fenugreek RNAseq data from the SRA database led to the identification of transcripts having extended sequence length and high homology to the contigs corresponding with the three significant SNPs, thus also for dDocent_Contig_39741_151 for which no-hit was identified in the NR database. For this contig, however, the matching transcript showed a lower degree of sequence homology than that observed for the previous contigs with their transcripts (Table S3). Translation of the TRINITY_DN2712_c0_g1_i2 transcript corresponding with dDocent_Contig_466 confirmed the presence of a TE, GAG, domain, and identifies another Zinc Finger DNA-binding domain of the C2HC type (ZnF-C2HC, Pfam: PF01530) downstream of the GAG domain. Translation of the TRINITY_DN59603_c0_g1_i1 transcript corresponding with contig dDocent_Contig_39741 identifies, slightly below the threshold value, a single long coiled-coil domain in which a Homeobox associated leucine zipper domain (HALZ, pfam: PF02183) is detected. Finally, translation of TRINITY_DN26743_c0_g1_i1 transcript which was identified for the dDocent_Contig_84790_24 confirms the presence of a retroviral integrase domain and identifies a short coiled-coil domain downstream. 

## 4. Discussion

Several sequencing technologies such as genotyping by sequencing [43], RAD-seq [17], and ddRAD-seq [18] have facilitated genotyping many genotypes with more SNP markers than was previously feasible. To explore a plant without a reference genome such as fenugreek, the ddRAD-seq was the technique of choice, as an inexpensive de novo sequencing technology, to generate a large number of SNP markers suitable for studying genetic diversity, population structure, and association mapping. Here, we report the first genetic diversity analysis of a fenugreek population consisting of 112 Egyptian genotypes genotyped with 38,142 high-quality polymorphic SNPs using the ddRAD-seq approach. Our study overcomes the limitations of using a limited number of genotypes and traditional dominant marker technologies such as RAPD, SRAP and SSR reported in earlier studies [12,13,14], which facilitates association mapping studies. The first step to identify a true marker-trait association is a detailed study of genetic diversity and population structure so that controls can be implemented to avoid false-positive associations [44]. The 38,142 SNPs were used for an in-depth understanding of the fenugreek population genetic diversity and structure to thoroughly infer how natural selection and/or plant breeding affected the formation and differentiation within the Egyptian fenugreek population. Results obtained from fastSTRUCTURE and the phylogenetic tree revealed two distinct main populations and six sub-populations. The presence of population structure in the examined collection was irrespective of their geographic origin. However, the admixture observed here meets our expectations and could be explained by seed exchange between farmers in local markets throughout the country over the long history of fenugreek cultivation in Egypt which is similar to what was reported recently in tea [45] and wheat [46]. 

As large seeds are expected to emerge more rapidly, to have greater seedling survival rate, and stress tolerance, we have chosen to study seed length and width. The high level of variation together with the relatively high heritability recorded for both traits suggested the suitability of our collection for association mapping studies and to effectively map the associated SNPs that can be further used in the fenugreek marker-assisted breeding programs. We employed MLM including population structure (Q), and kinship (K) matrix to avoid spurious associations. The two commonly used multiple comparison methods to select for the significant threshold level in association mapping studies are Bonferroni correction [37] and false discovery rate (FDR) [40]. In the present study and based on the calculated Bonferroni correction, the threshold was set to 6.5. We were able to map the same significant SNP, dDocent_Contig_466_145, to be associated with seed length and width with –log10(*P*) values of 6.7 and 8.4, and FDR of α = 0.007 and 0.000, respectively. However, using this very strict threshold would result in no significant SNPs associated with seed color. Earlier studies [47,48,49] have debated that Bonferroni correction for marker effects using both Q and K could result in over-correcting and the need to use a lower significance level of the *P*-value. Therefore, we checked the SNPs with −log10(*P*) values less than 6.5 and with FDR < 0.05. One SNP marker, dDocent_Contig_84790_24, was found to be associated with seed color at −log10(*P*) = 6.32. Considering the low FDR of this SNP, α = 0.019, and the high explained variance, 28%, of the observed variation, altogether, we believe this association can be considered as a true association. However, so far, no data is available which allows the alignment of the newly discovered markers in fenugreek to a comprehensive consensus map that covers its eight chromosomes [50,51]. Therefore, systematic targeting of those newly developed markers to homologous regions in other legumes could be the first step towards predicting the associated genes. 

A more in-depth analysis of the identified trait-associating SNPs shows that two SNPs are located in contigs containing sequences that are conserved among several species from the Trifolieae tribe. The close taxonomic relationship implied by this sequences conservation raises the prospect that the same genomic regions harboring the SNPs might be controlling the same traits among the species that belong to this tribe, permitting a translational approach to gene function discovery. However, further studies are required to confirm this hypothesis. Our finding that two SNPs associated with seed size (length and width) and seed color are both localized in TE genes could be explained by the fact that other species from the Trifolieae tribe, in contrast to other legumes, have genomes that are rich in TEs. For example, *Trifolium pretense* and *Trifolium medium* genomes were reported to have more than 30 and 40%, respectively, of retrotransposable elements in their genome [24,52], and that our data show a similar trend for the fenugreek genome. Accordingly, this suggests an important role for TEs in shaping the fenugreek genome and thus in controlling important phenotypic and economic traits in addition to the ones studied here. It is also possible that genes underlying seed traits such as seed size and color are maintained on neighbouring genomic regions as reported in other crop plants [6,53,54] and that the same region is highly populated with TEs. It is interesting to investigate this hypothesis once a full fenugreek genome draft becomes available.

Due to the existence of linkage disequilibrium, we are aware that it is not likely that the identified SNPs are indicating the exact genes underlying a certain trait and that they could rather be merely an association with the trait. However, it is still possible that once a SNP is located in an ORF of a gene, investigating the putative function of this gene and its functional domains can help accepting or rejecting the hypothesis regarding the direct involvement of this gene in the studied trait. In the current scenario, gene modeling analysis and the RNAseq expression data indicate that all the associating SNPs were localized in transcribed genes. In the case of the SNP associated with seed length and width, dDocent_Contig_466_145, it is possible that the identified Znf-C2HC domain, downstream of the GAG domain of the TE is playing a role in gene transcription and thus affecting seed size. This would be similar to what has been reported for the ZnF-C2H2 domain encoding gene that was identified in *M. truncatula* and which was found to affect seed size [55]. Our further investigation of the protein sequence suggests that it is a truncated form of a similar multi-domain homolog that is present in *Trifolium pretense* (PNX92211.1) which, similar to our SNP-related ORF, contains additional TE-related domains downstream of the Znf-C2HC domain. This strengthens the notion that this SNP could be in a gene that has a direct effect on seed size. For the other identified SNPs, associated with only seed width or seed color, it is however difficult to conclude whether the corresponding ORFs are affecting the respective trait. These results highlight the possible role of *M. truncatula* as well as the Trifolieae tribe as a proxy for gene content and order in the fenugreek genome. This is similar to previous studies that reported synteny between *M. truncatula* and legumes such as white clover [23], red clover [56], birdsfoot trefoil [57,58], common bean [58], chickpea and lentil [59], and faba bean [60]. Synteny was also reported between members of the Trifolieae tribe such as Red clover and zigzag clover [24].

## 5. Conclusions

Genotyping an Egyptian collection of 112 fenugreek genotypes with 38,142 SNPs using the ddRAD sequencing enabled us to investigate the genetic diversity and the population structure of our collection. Our results revealed that the population is divided into two main and 5 sub-populations. We used seed length, width and color to prove the suitability of this population for association mapping studies and we found three trait-associated SNPs. Our results indicated the possible role of *M. truncatula* and the Trifolieae tribe to improve our understanding to the fenugreek genome. Using such a well-genotyped collection to investigate more complex traits is the first step towards identifying the underlying genes. Further evaluating this population under diverse environmental conditions can help to dissect genotype by environment interactions and to improve fenugreek marker-assisted breeding programs.

## Figures and Tables

**Table 1:** Significant SNPs associated with seed length, width, and color of 112 fenugreek genotypes detected using mixed linear model. MAF = minor allele frequency, R^2^ = explained phenotypic variance, FDR = false discovery rate.

Trait	SNP	−LOG10 (P)	MAF	R^2^	FDR	Sequences of ddRAD Primers
Seed Length	dDocent_Contig_466_145	6.71	0.36	0.39	0.007	GAGACTGCTGAATTTTCCAAGTGTATTAAGTTTGAGAATGGTCTGCGTGC[T]GAGATTAAGTGGGCCATTGGGTACCAGAAGATCNNNNNNNNNNTAATTCT
Seed Width	dDocent_Contig_466_145	8.36	0.36	0.46	0.000	
	dDocent_Contig_39741_151	6.88	0.28	0.38	0.002	TTGAAGGTTGCTAAGGAGGGCGCTGGCTCGGCAGGTCCGAAGGAGACTGC[T]GAGATTGCCAGCCTCAGTCGCGCAGAGTTGATCNNNNNNNNNNAATTCTG
Seed Color	dDocent_Contig_84790_24	6.32	0.05	0.28	0.016	NAATTCTAACTCTTCCCGTAGTG[C]TGGCCCCCGTTCTCCAACTGAGTACGTTCATCTCGATTGGGATGACGGCC

## Supplementary Materials

The following are available online at https://www.mdpi.com/2073-4425/11/8/893/s1, Table S1: Geographic information of the 112 tested fenugreek genotypes; Table S2: Values of seed length, width, and color (RGB) and their corresponding qualitative values used for the association mapping; Table S3: Contig sequences, corresponding Trinity transcripts and translation into protein.
